# Differential impact of affective and cognitive attributes on preference under deliberation and distraction

**DOI:** 10.3389/fpsyg.2015.00549

**Published:** 2015-04-30

**Authors:** Zuo-Jun Wang, Kai-Qin Chan, Jiao-Jiao Chen, Ai Chen, Fei Wang

**Affiliations:** ^1^Department of Psychology, Ningbo UniversityNingbo, China; ^2^Department of Social and Cultural Psychology, Behavioral Science Institute, Radboud UniversityNijmegen, Netherlands; ^3^Department of Advertising, Xiamen UniversityXiamen, China

**Keywords:** deliberation, distraction, affective, cognitive, unconscious thought

## Abstract

Two experiments were designed to test the hypothesis that affective information looms relatively larger than cognitive information when individuals are distracted for a period of time compared to when they engage in deliberative thinking. In two studies, participants were presented with information about 4 decision alternatives: An *affective alternative* that scored high on affective attributes but low on cognitive attributes, a *cognitive alternative* with the opposite trade-off, and two fillers. They were then asked to indicate their attitudes toward each of four decision alternatives either immediately, after a period of deliberation, or after a period of distraction. The results of both experiments demonstrated that participants significantly preferred the affective alternative to the cognitive alternative after distraction, but not after deliberation. The implications for understanding when and how unconscious thought may lead to better decisions are being discussed.

## Introduction

When people make a choice, they often need to make tradeoffs between affective (hedonic) and cognitive (utilitarian) attributes. As an example, one may need to make a choice between a glass of soy or chocolate milk. Soy milk is healthy (a positive cognitive feature) but not all that tasty (a neutral or even negative affective feature), whereas chocolate milk is very tasty (a positive affective feature) but not very healthy (a negative cognitive feature). A substantial amount of research has established that the influence of these two types of features or attributes on preference or choice depends on the context (Wilson and Schooler, 1991; Wilson et al., 1993; Dhar and Wertenbroch, 2000; Dubé and Cantin, 2000). For instance, it was demonstrated that “food liking” is more influenced by the affective attribute (e.g., refreshing, tasteful) of a product, whereas “food consumption” is more influenced by the cognitive attribute (e.g., healthy, full of vitamins) (Dubé and Cantin, 2000), and that the relative salience of affective attributes is greater in forfeiture choices than in acquisition choices (Dhar and Wertenbroch, 2000).

Given that deliberatively thinking or distracting oneself (or being distracted) for some time are two usual ways for people to make a difficult choice, the central question we ask here is whether the influence of affective and cognitive attributes on preference depends on the two ways of making a choice. The answer to this question would shed some light on the underlying process of the effects that deliberation deteriorates (e.g., Wilson and Schooler, 1991; Wilson et al., 1993), while distraction improves decision quality or satisfaction (e.g., Dijksterhuis and Nordgren, 2006; Lerouge, 2009; Ham and van den Bos, 2010; Messner and Wänke, 2011; Strick et al., 2011; Abadie et al., 2013; Creswell et al., 2013). From a practical view, a better understanding of what information can largely influence and predict people's choice under deliberation and distraction has implications for everyday decision making, as well as for marketing and persuasive communication. We assume that affective attributes loom relatively larger after a period of distraction whereas cognitive attributes have more impact on preference after a period of deliberation. This assumption is derived from the following two lines of research.

One line of research demonstrates that deliberately thinking reduces quality or satisfaction of decisions (Wilson and Schooler, 1991; Wilson et al., 1993). In one of the studies, participants were asked to choose between art posters under a deliberation condition where they were asked to list their reasons for liking or disliking each poster, and a control condition where they were not given such an opportunity. All participants were given their selected art poster to take home and were phoned a few weeks later. Participants in the deliberation condition were less satisfied with their posters than control participants. The authors proposed that participants in the deliberation condition may put too much weight on attributes that are easy to verbalize and seem like plausible reasons but put less weight to feelings which are often difficult to be verbalized exactly. Over time, however, when their true attitudes return, they come to feel less satisfied (Wilson et al., 1993). In line with this view, Bohm and Pfister (1996) asked participants to indicate their preference for a set of options (i.e., eight prizes they had won and could choose from), as well as rate utilitarian and affective associations of these options, either in a private choice context (i.e., in a mail lottery) or in a public choice context (i.e., in a TV show). The authors show that in the private choice context, participants' preferences were more influenced by affective associations, whereas in a public choice context, due to justification demands, preferences were more influenced by utilitarian considerations.

Another line of research suggests that distraction period elicits unconscious thought, which leads to better complex decision quality (Lerouge, 2009; Ham and van den Bos, 2010; Strick et al., 2011), higher choice satisfaction (Dijksterhuis and van Olden, 2006; Messner and Wänke, 2011), as well as better lie detection (Reinhard et al., 2013). Unconscious thought refers to “object-relevant or task-relevant cognitive or affective thought processes that occur while conscious attention is directed elsewhere” (Dijksterhuis and Nordgren, 2006, p. 96). Regarding to the underlying processes of unconscious thought effect, Dijksterhuis and Nordgren (2006, p. 107) contended that the superior decision performance of unconscious thought might be because it “somehow uses the affective tone of the information better than conscious thought does.” Messner and Wänke (2011) found that participants are more satisfied with a praline chosen from a large assortment when they were distracted before choosing than when they either deliberated intensively or chose immediately. With regard to the possible explanation for this effect, in addition to the larger capacity of unconscious thought compared to conscious thought, the authors proposed that the higher choice satisfaction of unconscious thinkers might be caused by a greater reliance on the affective cues of a product (i.e., imaging the taste of a praline) whereas conscious thinkers may have relied too much on the individual components and neglected to imagine how these components would taste in combination. According to these propositions, it seems that affective attributes that activate more feelings and affective considerations will loom relatively larger when people engage in unconscious thought than when they engage in deliberately thinking.

Based on the above analysis, the present research tested the hypothesis that affective attributes loom relatively larger under a period distraction whereas cognitive attributes will have more impact after a period deliberation. In two experiments, participants were presented with information about four hypothetical alternatives (i.e., apartments). One alternative was designed as the *affective* alternative that is superior on affective attributes relative to cognitive attributes. Another apartment was designed as the *cognitive* alternative that is superior on cognitive attributes relative to affective attributes. The remaining two alternatives were set as fillers. We predict that participants who were distracted for a period of time will show a relatively stronger preference for the affective alternative than those who engaged in deliberatively thinking.

It should be noted in advance that we do not aim to show that one mode of thought leads to better results than the other mode of thought. We focus on affective vs. cognitive attributes, and whether affective or cognitive attributes make one alternative better than another alternative depends entirely on the decision domain and on the specific attribute in question, a point which we return to in the Discussion.

## Experiment 1

### Methods

#### Participants and design

One hundred and eighty students recruited on the campus (150 female, 30 male, mean age = 21.20 years) were randomly assigned to a distraction, a deliberation, and an immediate condition (60 participants per condition). Participants were paid a small fee for their participation^1^.

#### Materials

In the study, we described the four apartments (Apartments A–D) in terms of two affective attributes and two cognitive attributes^2^. Alternatives differed in the valence of these two types of attributes. Apartment A and C were filler alternatives. Apartment B had high values on the affective attributes (e.g., view of the apartment is a park) but low values on the cognitive attributes (e.g., 20 min away from the work or study place) was designed and was termed the *affective alternative*. Apartment D offered the opposite trade-off, namely, with low values on the affective attributes (e.g., view of the apartment is a parking lot) but high values on the cognitive (e.g., 10 min away from the work or study place) was termed the *cognitive alternative*. Table 1 shows complete description of the alternatives.

**Table 1:** **Attributes of apartments in Experiment 1**.

	**Affective attributes**		**Cognitive attributes**	
	**View from the apartment**	**Landlord**	**Security level**	**Distance to work or study**
Apartment A (filler)	Park	Unkind	Above average	20 min
Apartment B (affective)	Park	Kind	Average	20 min
Apartment C (filler)	Parking lot	Kind	Average	10 min
Apartment D (cognitive)	Parking lot	Unkind	Above average	10 min

#### Procedure

The experiment was described as an experiment on decision-making. Participants were presented with information about four hypothetical apartments, labeled A, B, C, and D, respectively. They were asked to form an impression of the four apartments. The attributes of each apartment were presented, one by one in random order (4 s per attribute). The order of presentation of information about the four apartments was randomized.

Thereafter, participants were randomly assigned to one of three conditions. In the immediate condition, they were immediately asked to give their attitude toward each of the four apartments (in the order from Apartments A to D) on 11-point scales ranging from 0 (*extremely negative*) to 10 (*extremely positive*). Participants in the deliberation condition were asked to “think very carefully about what you think of each of the four apartments and write down the advantages and disadvantages of the apartments.” In the distraction condition, after the presentation of the four apartments, participants were presented with a series of digital numbers (ranging from 1 to 99) and for each number they were asked to decide whether it is a multiple of 3 or not as quickly as possible by pressing a key. Three minutes later, participants in both the deliberation and distraction conditions were asked to respond to the same attitude measure, as participants in the immediate condition.

### Results

A 2 (type of alternative: affective alternative/cognitive alternative)^3^ × 3 (decision condition: distraction/deliberation/immediate condition) mixed ANOVA showed a significant main effect of the type of alternative, *F*_(1, 177)_ = 30.59, *p* < 0.01, η^2^*_p_* = 0.147, no main effect of decision condition, *F*_(2, 177)_ = 1.04, *p* = 0.62, η^2^_*p*_ = 0.005, and a significant interaction between the type of alternative and the decision condition, *F*_(2, 177)_ = 3.15, *p* < 0.05, η^2^_*p*_ = 0.034.

**Figure 1:**
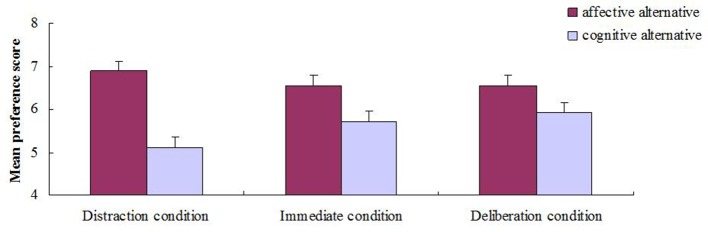
**Average preference score for affective and cognitive alternatives as a function of decision condition**. Error bars represent 1 SEM.

Further analysis revealed a significant difference between the preference score for the affective alternative (*M* = 6.88, *SD* = 1.91) and the cognitive alternative (*M* = 5.12, *SD* = 1.75) in the distraction condition, *t*_(59)_ = 5.14, *p* < 0.001, *d* = 0.96. The difference between the preference score for the affective alternative (*M* = 6.55, *SD* = 2.00) and the cognitive alternative (*M* = 5.70, *SD* = 1.86) was also significant in the immediate condition, *t*_(59)_ = 2.48, *p* < 0.05, *d* = 0.44. However, no significant difference was found between the preference score for the affective alternative (*M* = 6.55, *SD* = 1.65) and the preference score for the cognitive alternative (*M* = 5.92, *SD* = 1.92) in the deliberation condition, *t*_(59)_ = 1.92, *p* > 0.05, *d* = 0.35. Participants' mean preference score for the alternatives is graphed as a function of decision condition in Figure 1.

The immediate condition was designed as a baseline to further determine whether it is deliberation or thought process that occurs during distraction, or both, that influenced the preference. Of particular interest is whether distraction leads to preference change compared to immediate condition. If this is true, it might indicate that distraction elicits some special thought process (i.e., unconscious thought) as suggested by Dijksterhuis (2004), Dijksterhuis and Nordgren (2006).

We therefore firstly compared the preference pattern between the immediate and distraction conditions. A 2 (type of alternative: cognitive/affective) × 2 (decision condition: distraction/immediate) mixed ANOVA revealed a marginally significant interaction [*F*_(1, 118)_ = 3.56, *p* = 0.06, η^2^*_p_* = 0.03]. A Bayesian approach (Dienes, 2008, 2014) was taken to interpret this finding. The raw interaction effect between these two conditions is: (preference for affective alternative - preference for cognitive alternative) in the distraction condition - (preference for affective alternative - preference for cognitive alternative) in the immediate condition = (6.88 − 5.12) − (6.55 − 5.70) = 0.91. To calculate Bayes factor, the predicted size of the interaction needs to be specified, based on a specific theoretical framework. If distraction leads to more weight to affective attributes than cognitive attributes, while deliberation works the opposite way, with immediate condition falling in between, then the maximum size of the interaction effect between the distraction and immediate conditions would be the interaction effect between the distraction and deliberation conditions. That is, (preference for affective alternative - preference for cognitive alternative) in the distraction condition - (preference for affective alternative - preference for cognitive alternative) in the deliberation condition = (6.88 − 5.12) − (6.55 − 5.92) = 1.13. The interaction effect would not be smaller than 0.

Bayes factor (*B*) calculated using the online software described in Dienes (2008), representing the predictions as uniform distribution, is 4.16. *Bs* less than 1/3 indicate substantial support for the null, *Bs* between 3 and 10 indicate substantial support for the alternative (*Bs* above 10 indicate “strongly” support for alternative), and *Bs* between 1/3 and 3 indicate data insensitive (Jeffreys, 1939/1961). Therefore, the results provided substantial supporting evidence for the hypothesis that distraction leads to more weight being given to the affective attributes than cognitive attributes.

Second, to compare the preference pattern between the immediate and deliberation conditions, the interaction between these two conditions were analyzed using a 2 (type of alternative: cognitive/affective) × 2 (decision condition: immediate/deliberation) mixed ANOVA. Non-significant difference was obtained [*F*_(1, 118)_ = 0.21, *p* = 0.65, η^2^*_p_* = 0.00]. Bayes analysis was performed based on the similar rationale as above. Bayes factor (*B* = 0.74) indicate data insensitivity.

### Discussion

These findings support the hypothesis that affective attributes weigh more strongly than cognitive attributes under distraction than deliberation. Bayesian analysis indicates that distraction may elicit unconscious thought, which leads to different preference pattern between the distraction condition and immediate condition.

One may note that in all conditions, the affective alternative was more positive relative to the cognitive alternative (though not significantly so in the deliberation condition). In principle, it should be possible to find a preference reversal in that participants who were in the distraction condition preferred the affective alternative over the cognitive alternative (as found in Experiment 1) and that participants who engaged in deliberation preferred the cognitive alternative over the affective alternative. In order to increase the possibility of finding this reversal, we changed our stimulus materials in Experiment 2.

## Experiment 2

Experiment 2 aimed at extending the results of Experiment 1 with the following three aspects changed. First, the number of attributes of each alternative was increased from 4 to 6 such that the decision task was relatively more complex, and we added an important cognitive aspect (i.e., cost) to make the cognitive alternative more attractive. Second, we presented the attributes of each apartment as a list and made them appear all at once rather than one by one in random order. Third, a thought process check was employed to confirm the effectiveness of the manipulation of the deliberation and distraction conditions.

### Methods

#### Participants and design

One hundred and eighty students recruited on campus (128 female, 52 male, mean age = 20.82 years) were randomly assigned to one of three conditions: a distraction, a deliberation, and an immediate condition (60 participants per condition). They were paid a small fee for their participation.

#### Materials

As in Experiment 1, Apartment B was designed as the affective alternative which had high values on the affective attributes but low values on the cognitive attributes and Apartment D was designed as the cognitive alternative which had low values on the affective attributes but high values on the cognitive attributes. Apartments A and C were fillers. Table 2 shows the complete description of the alternatives.

**Table 2:** **Attributes of apartments in Experiment 2**.

	**Affective attributes**			**Cognitive attributes**		
	**View from the apartment**	**Residential landscaping**	**Landlord**	**Rent for the apartment**	**Distance to work or study**	**Network signal**
Apartment A (filler)	Park	Pleasant	Unkind	Relatively expensive	Relatively close	Poor
Apartment B (affective)	Park	Pleasant	Kind	Relatively expensive	Relatively far	Poor
Apartment C (filler)	Parking lot	Unpleasant	Kind	Relatively cheap	Relatively far	Strong
Apartment D (cognitive)	Parking lot	Unpleasant	Unkind	Relatively cheap	Relatively close	Strong

#### Procedure

The attributes of each apartment were presented as a list and appeared all at once for 15 s. Furthermore, a manipulation check of the thought process was included in the deliberation and distraction conditions. Participants were asked to respond to an item “Have you engaged in deliberation about the information about the apartments?” (0 = “*not at all*”; 10 = “*very often*”).

### Results

#### Manipulation check

The score in the deliberation condition (*M* = 7.32, *SD* = 1.88) was significantly higher than that in the distraction condition (*M* = 4.20, *SD* = 3.12) on thought process check (*t* = −6.63, *p* < 0.001), which confirmed the effectiveness of the manipulation.

#### Preference

A 2 (type of alternative: affective alternative/cognitive alternative) × 3 (decision condition: distraction/deliberation/immediate condition) mixed repeated ANOVA showed no significant main effects of type of alternative, *F*_(1, 177)_ = 1.09, *p* = 0.30, η^2^*_p_* = 0.01, and decision condition, *F*_(2, 177)_ = 0.57, *p* = 0.57, η^2^*_p_* = 0.01, but a significant interaction, *F*_(2, 177)_ = 7.68, *p* < 0.01, η^2^*_p_* = 0.08.

**Figure 2:**
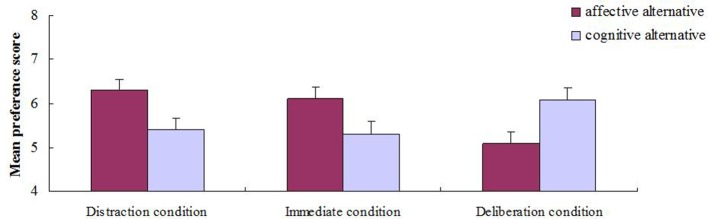
**Average preference score for affective and cognitive alternatives as a function of decision condition**. Error bars represent 1 SEM.

Further analysis revealed that the preference score for the affective alternative (*M* = 6.30, *SD* = 2.04) was significantly higher than that for the cognitive alternative (*M* = 5.43, *SD* = 1.98) in the distraction condition, *t*_(59)_ = 2.36, *p* < 0.05, *d* = 0.43. The difference between the preference score for the affective alternative (*M* = 6.12, *SD* = 2.00) and the cognitive alternative (*M* = 5.32, *SD* = 1.97) was also significant in the immediate condition, *t*_(59)_ = 2.12, *p* < 0.05, *d* = 0.39. However, a clearly reversed pattern was observed in the deliberation condition, that is, the preference score for the cognitive alternative (*M* = 6.08, *SD* = 2.33) was significantly higher than that for the affective alternative (*M* = 5.10, *SD* = 1.90), *t*_(59)_ = −2.52, *p* < 0.05, *d* = 0.46. Participants' mean preference score for the alternatives is graphed as a function of decision condition in Figure 2.

As in Experiment 1, preference pattern for the affective and cognitive alternatives in the distraction and deliberation conditions was compared to that in the immediate condition respectively. A 2 (type of alternative: cognitive/affective) × 2 (decision condition: distraction/immediate) mixed ANOVA revealed a non-significant interaction between the distraction condition and immediate condition, *F*_(1, 118)_ = 0.02, *p* = 0.90, η^2^*_p_* = 0.00. The raw interaction effect between these two conditions is 0.07. The predicted size of interaction effect ranges from 0 to 1.85. Bayes factor (*B* = 0.37) indicates data insensitivity. A 2 (type of alternative: cognitive/affective) × 2 (decision condition: immediate/deliberation) mixed ANOVA revealed a significant interaction between the immediate and deliberation conditions, *F*_(1, 118)_ = 10.78, *p* = 0.001, η^2^*_p_* = 0.08. Bayes factor (*B* = 80.78) provides strong evidence for the hypothesis that a period of deliberation attached more weight to cognitive attributes than affective attributes.

### Discussion

These findings strongly supported the hypothesis that affective attributes weigh stronger than cognitive attributes under the distraction condition compared to the deliberation condition, which led to the preference for the affective alternative in the distraction condition but preference for the cognitive alternative in the deliberation condition.

## General discussion

The present study examined whether affective and cognitive attributes would disproportionately influence preferences under deliberation and distraction. Indeed, we found that distraction, compared to deliberation, led people to put more weight on affective rather than cognitive attributes. Comparing the deliberation, as well as the distraction condition, to the immediate condition provides a chance to further determine whether it is distraction or deliberation that influenced the salience of the affective and cognitive attributes. The findings in Experiment 1, based on Bayes analysis, provide substantial evidence for the hypothesis that distraction influenced the relative salience of the affective and cognitive attributes, although it was not confirmed in Experiment 2 because of data insensitivity. The findings in Experiment 2 provide strong evidence for the hypothesis that cognitive attributes have more impact on preference after a period of deliberation. Taken together, these findings indicate that both distraction and deliberation influence the salience of the affective and cognitive attributes.

Different preference pattern between the distraction condition and immediate condition seems indicate that some special thought process—perhaps unconscious thought as defined by Dijksterhuis (2004), Dijksterhuis and Nordgren (2006)—occurred during the distraction period. If not, no difference would have been observed between these two conditions. Then, why are affective alternatives preferred by unconscious thought? Unconscious thought is described as a holistic process that gives rough estimates (Dijksterhuis and Nordgren, 2006). Recently, Abadie et al. (2013) suggest that unconscious thought is more likely to be based on gist representations rather than verbatim representations. An effective response to an alternative formed mainly by affective attributes, such as kind vs. unkind landlord, is a holistic, qualitative or vague representation of the alternative. Alternatively, a cognitive response, which mainly formed by cognitive attributes such as the distance to work is 10 vs. 20 min, is relatively more precise and quantitative. That may be why the influence of affective attributes on preference was relatively unaffected during distraction or unconscious thought. Creswell et al. (2013) provided the first neural evidence for the unconscious thought process. They found that the same brain regions (the right dorsolateral prefrontal cortex and left intermediate visual cortex) that were responsible for information encoding continue to be active during distraction (i.e., unconscious thought), and the strength of these activations predicted the ability to discriminate between best and worst items in the decision tasks that the participant undertook. Visual cortex activation is associated with vividness of perception or memories. The vividness of information is to some extent depending on its affective property (McGill and Anand, 1989; Bywaters et al., 2004; Todd et al., 2012). Affective attributes, therefore, maintain its salience during unconscious thought processes.

It should be noted, however, that, as we briefly hinted at in the Introduction, our studies were not aimed at showing that one of the modes of thought (conscious vs. unconscious thought) leads to better decisions than the other. Actually, much doubt has been cast in recent years on the unconscious thought advantage (see Acker, 2008; Nieuwenstein and Van Rijn, 2012; Nieuwenstein et al., 2015). We focused on whether affective or cognitive attributes have different influence on preference under distraction and deliberation. Another point that need to be noted is that merely stating a preference without having to experience the consequences of that choice feels quite different compared to deciding for something and then having to live with the potential consequences of the decision. It would be interesting in future research to investigate choice tasks with real consequences.

Although our studies did not focus on decision quality or satisfaction, it may still have implications for making a better choice. Previous research on attitude-behavior relation suggests that behaviors may either be cognitively or affectively driven by attitudes, and thought processes may make either the affective or cognitive components of the attitude more salient and thus more important when making choices. A match between the attitude components that drive behavior and the attitude components emphasized by thought processes would increase the attitude-behavior relation, whereas a mismatch would decrease the relation (Millar and Tesser, 1986; Wilson and Dunn, 1986). If choices are affectively driven—one can think of (romantic) relationships, or art, in this respect—let affective attributes play a larger role than cognitive attributes would improve decision satisfaction (i.e., attitude-behavior relation). On other occasions, such as when buying a new washing machine, it seems reasonable to let the cognitive attributes trump the affective ones. And with yet other decisions—such as when buying a house—both should ideally be taken into account. If our conclusions are confirmed in independent replications, the current analysis may lead to a very practical moderator: When a decision is largely affective, make it after a period of distraction, if it is very cognitive, engage in deliberation first.

### Conflict of interest statement

The authors declare that the research was conducted in the absence of any commercial or financial relationships that could be construed as a potential conflict of interest.

## Footnotes

^1^This research was conducted in accordance with the ethical guidelines of the American Psychological Association and approved by the academic and ethics committee of Department of psychology, Ningbo University. We determined our sample size by basing it on the central limit theorem. We sampled until we had 60 per condition (our stopping rule).

^2^In the Online Supplementary Information, we describe how these attributes were chosen. From the chosen attributes, we then validated the distinctiveness of the affective attributes from the cognitive attributes.

^3^For each of the three conditions, the preference scores for the two fillers fell somewhere in between the two experimental alternatives (Apartments B and D) in both Experiments 1 and 2.

## References

[B1] Abadie M. Waroquier L. Terrier P. (2013). Gist memory in the unconscious-thought effect. Psychol. Sci. 24, 1253–1259. 10.1177/0956797612470958 23698616

[B2] Acker F. (2008). New findings on unconscious versus conscious thought in decision making: additional empirical data and meta-analysis. Judgm. Decis. Mak. 3, 292–303.

[B3] Bohm G. Pfister H. R. (1996). Instrumental or emotional evaluations: what determines preferences? Acta Psychol. Amst). 93, 135–148. 10.1016/0001-6918(96)00017-0 8826793

[B4] Bywaters M. Andrade J. Turpin G. (2004). Determinants of the vividness of visual imagery: the effects of delayed recall, stimulus affect and individual differences. Memory 12, 479–488. 10.1080/09658210444000160 15487543

[B5] Creswell J. D. Bursley J. K. Satpute A. B. (2013). Neural reactivation links unconscious thought to decision-making performance. Soc. Cogn. Affect. Neurosci. 8, 863–869. 10.1093/scan/nst004 23314012 PMC3831563

[B6] Dhar R. Wertenbroch K. (2000). Consumer choice between hedonic and utilitarian goods. J. Mark. Res. 27, 60–71 10.1509/jmkr.37.1.60.18718

[B7] Dienes Z. (2008). Understanding Psychology as a Science: An Introduction to Scientific and Statistical Inference. Basingstoke: PalgraveMacmillan.

[B8] Dienes Z. (2014). Using Bayes to get the most out of non-significant results. Front. Psychol. 5:781. 10.3389/fpsyg.2014.00781 25120503 PMC4114196

[B9] Dijksterhuis A. (2004). Think different: the merits of unconscious though in preference development and decision making. J. Pers. Soc. Psychol. 87, 586–598. 10.1037/0022-3514.87.5.586 15535773

[B10] Dijksterhuis A. Nordgren L. F. (2006). A theory of unconscious thought. Perspect. Psychol. Sci. 1, 95–109 10.1111/j.1745-6916.2006.00007.x 26151465

[B11] Dijksterhuis A. van Olden Z. (2006). On the benefits of thinking unconsciously: unconscious thought increases post-choice satisfaction. J. Exp. Soc. Psychol. 42, 627–631 10.1016/j.jesp.2005.10.008

[B12] Dubé L. Cantin I. (2000). Promoting health or promoting pleasure? A contingency approach to the effect of informational and emotional appeals on food liking and consumption. Appetite 35, 251–262. 10.1006/appe.2000.0361 11073707

[B13] Ham J. van den Bos K. (2010). On unconscious morality: the effects of unconscious thinking on moral decision making. Soc. Cogn. 28, 74–83 10.1521/soco.2010.28.1.74

[B14] Jeffreys H. (1939/1961). The Theory of Probability, 1st/3rd Edn. Oxford: Oxford University Press.

[B15] Lerouge D. (2009). Evaluating the benefits of distraction on product evaluations: the mindset effect. J. Consum. Res. 36, 367–379 10.1086/599047

[B16] McGill A. L. Anand P. (1989). The effect of vivid attributes on the evaluation of alternatives: the role of differential attention and cognitive elaboration. J. Consum. Res. 16, 188–196 10.1086/209207

[B17] Messner C. Wänke M. (2011). Unconscious information processing reduces information overload and increases product satisfaction. J. Consum. Psychol. 21, 9–13 10.1016/j.jcps.2010.09.010

[B18] Millar M. G. Tesser A. (1986). Effects of affective and cognitive focus on the attitude-behavior relation. J. Pers. Soc. Psychol. 51, 270–276 10.1037/0022-3514.51.2.270

[B19] Nieuwenstein M. R. Van Rijn H. (2012). The unconscious thought advantage: further replication failures from a search for confirmatory evidence. Judgm. Decis. Mak. 7, 779–798.

[B20] Nieuwenstein M. R. Wierenga T. Morey R. D. Wicherts J. M. Blom T. N. Wagenmakers E. J. (2015). On making the right choice: a meta-analysis and large-scale replication attempt of the unconscious thought advantage. Judgm. Decis. Mak. 10, 1–17.

[B21] Reinhard M. A. Greifeneder R. Scharmach M. (2013). Unconscious processes improve lie detection. J. Pers. Soc. Psychol. 105, 721–739. 10.1037/a0034352 24219784

[B22] Strick M. Dijksterhuis A. Bos M. W. Sjoerdsma A. Van Baaren R. B. Nordgren L. F.. (2011). A meta-analysis on unconscious thought effects. Soc. Cogn. 29, 738–762. 10.1521/soco.2011.29.6.73819379025

[B23] Todd R. M. Talmi D. Schmitz T. W. Susskind J. Anderson A. K. (2012). Psychophysical and neural evidence for emotion-enhanced perceptual vividness. J. Neurosci. 32, 11201–11212. 10.1523/JNEUROSCI.0155-12.2012 22895705 PMC3449277

[B24] Wilson T. D. Dunn D. S. (1986). Effects of introspection on attitude-behavior consistency: analyzing reasons versus focusing on feelings. J. Exp. Soc. Psychol. 22, 249–263 10.1016/0022-1031(86)90028-4

[B25] Wilson T. D. Lisle D. J. Schooler D. Hodges S. D. Klaaren K. LaFleur S. J. (1993). Introspecting about reason can reduce post-choice satisfaction. Pers. Soc. Psychol. Bull. 19, 331–339 10.1177/0146167293193010

[B26] Wilson T. D. Schooler J. W. (1991). Thinking too much: introspection can reduce the quality of preferences and decisions. J. Pers. Soc. Psychol. 60, 181–192. 10.1037/0022-3514.60.2.181 2016668

