# Supplementary material for: Differential impact of affective and cognitive attributes on preference under deliberation and distraction
Source: Front Psychol. 2015 Apr 30;6:549. doi: 10.3389/fpsyg.2015.00549 (PMC4415306; doi:10.3389/fpsyg.2015.00549)
Supplement: Supplementary file 1 [file DataSheet1.DOC]

**Online Supplementary Information**

**Pilot for Experiment 1**

We sought to identify affective and cognitive attributes for use in Experiment 1. An independent sample of 30 participants was asked to consider some attributes when renting an apartment. They were presented with 24 apartment attributes, for instance, “view from apartment (park vs. parking lot)” and “landlord (unkind vs. kind)”, etc.. They were asked to rate the “ease of evoking affect” and the “importance” of each attribute on 5-point scales, ranging from *quite difficult to evoke affect* (1) to *very easy to evoke affect* (5) and from *not at all important* (1) to *extremely important* (5).

Of the 24 attributes, the two attributes “view from apartment (park vs. parking lot)” and “landlord (unkind vs. kind)” were chosen and termed as affective attributes because they were rated relatively higher in easy of evoking affect but lower in importance. The other two attributes “security level (above the average vs. average)” and “distance to work or study (20 minutes vs. 10 minutes)” were chosen and termed as cognitive attributes because they were rated lower in easy of evoking affect but relatively higher in importance. Mean rating of ease of evoking affect combined by the two affective attributes was significantly higher (*M* = 4.28, *SD* = .83) than that combined by the two cognitive attributes (*M* = 3.58, *SD* = .88, *p* < .01), whereas mean importance rating combined by the two affective attributes (*M* = 4.0, *SD* = .77) was significantly lower than that combined by the two cognitive attributes (*M* = 4.37, *SD* = .54, *p* < .05).

**Pilot for Experiment 2**

This pilot study is similar to the previous pilot study, except that 18 attributes (some of which were not in the previous pilot study) were presented to an independent sample of 35 participants. Of the 18 attributes, “residential landscaping (pleasant vs. unpleasant)”, “view from apartment (park vs. parking lot)” and “landlord (kind vs. unkind)” were chosen as affective attributes, and “network signal (strong vs. poor)”, “rent for the apartment (relatively cheap vs. relatively expensive)” and “distance to work or study (relatively close vs. relatively far)” were chosen as cognitive attributes. Mean rating of ease of evoking affect combined by the three affective attributes was significantly higher (*M* = 4.07, *SD* = .73) than that combined by the three cognitive attributes (*M* = 3.71, *SD* = .93, *p* = .026), whereas mean importance rating combined by the three affective attributes (*M* = 3.84, *SD* = .71) were significantly lower than that combined by the three cognitive attributes (*M* = 4.24, *SD* = .63, *p* = .003).

**Confirming the distinction between the chosen Affective and Cognitive Attributes**

It is possible that participants’ understanding of the concept of “importance” did not map perfectly to our concept of “cognitive attributes”. For example, an affective attribute may also be considered important. Therefore, we conducted a confirmatory study to validate the distinctiveness of our set of affective and cognitive attributes.

An independent sample of 30 participants were asked to rate whether each attribute identified in the previous two pilot studies (see below) is perceived to be more of an affective attribute or a cognitive attribute on a 7-point scale ranging from -3 (*cognitive attribute*) to +3 (*affective attribute*). They were also clearly informed that *affective attribute* refers to attribute that activates more feelings or affective associations about the target, whereas *cognitive attribute* refers to attribute that activates more instrumental or functional considerations.

The results confirmed that the affective attributes were rated as more affective when compared to zero (i.e., the midpoint of the scale). The opposite was true for the cognitive attributes. All *p*_s_ < .01 (see Table 3). Therefore, in Experiments 1 and 2, the chosen affective and cognitive attributes were clearly distinctive from each other on the affect-cognition dimension.

| **Table 3. Means (standard deviations) of affective and cognitive attributes on cognitive-affective attribute scale** | | |
| --- | --- | --- |
| Affective attributes | view from the apartment  (park vs. parking lot) | 1.40 (1.65)^***^ |
|  | landlord  (kind vs. unkind) | 1.47 (1.83)^***^ |
|  | residential landscaping  (pleasant vs. unpleasant) | 1.67 (1.94)^***^ |
| Cognitive attributes | security level  (above the average vs. average) | -1.20 (2.30)^**^ |
|  | distance to work or study  (20 minutes vs. 10 minutes) | -1.77 (1.59)^***^ |
|  | rent for the apartment  (relatively expensive vs. relatively cheap) | -1.57 (1.85)^***^ |
|  | distance to work or study  (relatively far vs. relatively close) | -1.93 (1.26)^***^ |
|  | network signal  (poor vs. strong) | -1.37 (1.80)^***^ |

*Note*: ^**^ *p* < 0.01; ^***^ *p* < 0.001.
